# Quality of life in multiple scenarios: The impact of work mode and social contact quantity

**DOI:** 10.3389/fpsyg.2023.1018415

**Published:** 2023-01-24

**Authors:** Cheng-Han Leng, Chi-Shiun Tsai, Ta-Chien Chan, Hsuan-Wei Lee

**Affiliations:** ^1^Department of Psychology, National Taiwan University, Taipei City, Taiwan; ^2^Heinz College of Information Systems and Public Policy, Carnegie Mellon University, Pittsburgh, PA, United States; ^3^Research Center for Humanities and Social Sciences, Academia Sinica, Taipei City, Taiwan; ^4^Institute of Sociology, Academia Sinica, Taipei City, Taiwan

**Keywords:** work from home, quality of life, COVID-19, item response theory, differential item functioning, social contact

## Abstract

**Introduction:**

During the COVID-19 pandemic, many employees were encouraged to temporarily work from home as an attempt to decrease social contact with others. However, the employees' quality of life (QoL) may have been threatened by this mode of working. This study, therefore, aims to explore the employees' QoL given the new mode of working from home (WFH) as a result of the pandemic vs. working in the office (WIO), the amount of social contact that they were exposed to, and the ratio of face-to-face contact that they had.

**Methods:**

A total of 803 WFH employees and 588 WIO employees' QoL was assessed during the same time period using the WHOQOL-BREF, which contains four domains: physical health, psychological health, social relationship, and the environment. We then divided the participants into 16 groups in accordance with the levels of work mode, social contact quantity, and face-to-face contact ratio–forming a case-control study. A differential item functioning (DIF) analysis was used to analyze the responses on the WHOQOL-BREF under the 4-dimensional rating scale model fitting.

**Results:**

The results indicated that WFH employees' QoL was superior to that of WIO employees. The relationship between the WFH mode and the employees' QoL was specifically moderated by the amount of social contact and the ratio of face-to-face contact that was experienced. The results further demonstrated that the increased amount of non-face-to-face contact was better for WFH employees' QoL than that of WIO employees.

**Discussion:**

In conclusion, the WFH mode was practical during the COVID-19 pandemic, as our findings indicated that WFH employees' QoL was better than WIO employees' QoL. However, maintaining social connections is equally important as this allows employees to perform better at their jobs and maintain such performance. The employees with a higher number of social support had a better QoL. Additionally, the facets detected as DIF items provided implications for the QoL with regard to the research methodology and insight into factors affecting the employees' QoL.

## Introduction

According to The WHOQOL Group (1994, 1997), quality of life (QOL) is defined as a self-evaluation of an individual's physical health, psychological health, social relationships, and their environment in the last 2 months. For employees, thriving at work is associated with their QoL (Ventegodt, 1995, 1996). In addition, Rabianski (2007) indicates the importance of the employees' QoL as it mediates the relationship between job demands and organizational outcomes. The QoL can therefore be used as a basis for the profession of organizational management to evaluate the performance of employees.

The COVID-19 virus has swept the world from early 2020, causing millions of confirmed cases as well as deaths, and as a result, was acknowledged as a pandemic on March 11, 2020, by the WHO (2020). To limit spreading the virus, a social distancing rule was implemented and has been promoted to ensure that people maintain a recommended distance between one another. For employees, one of the policies implemented is to “work from home (WFH),” which means employees temporarily perform their duties virtually, from home– if it is possible. When compared to “work in the office (WIO),” WFH has a few advantages, namely: flexible working hours and saving time with regards to the amount of time spent commuting to work; however, there is difficulty with regards to separating home affairs from professional ones, social isolation, and being able to meet more significant organization requirements (Klopotek, 2017). The effective promotion of WFH has therefore been a challenge in the organizational management profession (Praptana and Riyanto, 2020; Wong et al., 2020). Whether or not employees are in favor of the WFH policy implemented, many employees could continue having a job that enables them to work from home. Several core businesses, however, could also continue operating and providing essential services. The WFH employees' social connections, however, reduced as a result of the WFH policy being implemented (Dockery and Bawa, 2020). As a result of the WFH policy being enacted to promote the social distancing rule and prevent social contact among people, the effectiveness of this policy may be moderated by the number of social connections recorded over a specified period. Sommerlad et al. (2021) suggested that people that are highly social had more depressive symptoms during an enforced reduction in social contact with others during the pandemic. In addition, older individuals' emotional well-being and loneliness were found to be adversely affected by the pandemic (Macdonald and Hülür, 2021), and WFH employees were reported to experience loneliness (Killgore et al., 2020; Riski et al., 2022). A recent study therefore demonstrated that the WFH policy was related to mental health disorders as a result of having to maintain social distancing from other (Marroquín et al., 2020). However, with the improvement of communication technology, people should still be easily connected by making use of the internet, as many people looked for health-related information, including social support on the internet (Freeman et al., 2008). In fact, as Litwin and Levinsky (2022)'s findings suggest, non-face-to-face social contact increased mental health problems, while face-to-face social contact decreased the problems. This suggested that the effect of social connections on the relationship between the WFH policy and the QoL may also be moderated by the contact method, e.g., face-to-face contact vs. non-face-to-face contact. There are a few studies, however, that have conducted research with regards to whether there is a difference in the WFH employees' QoL if an increased number of social contacts were conducted in different contact methods. We therefore considered not only the number of social connections but also the ratio of face-to-face contact while studying the WFH employees' QoL during the pandemic.

In this study, we study the effectiveness of WFH by assessing the WFH employees' QoL, owing to the fact that the employees' performance is related to their QoL. To assess the QoL, the WHOQOL-BREF, developed by the WHOQOL group initiated by fifteen international field centers (The WHOQOL Group, 1996), was recommended for this study. The invariance of the WHOQOL-BREF in measuring the QoL of both the WIO and WFH employees has yet to be discovered. Owing to the fact that the WHOQOL-BREF was developed based on general and clinical respondents (The WHOQOL Group, 1996), some items in the WHOQOL-BREF might assess the employees' QoL with bias. For instance, for an item that measures the high-level QoL, typically, people with a high-level QoL should be able to answer higher-level options. However, when many respondents with a low-level QoL respond with high-level ratings, the item may have measurement bias. For this reason, in evaluating the fitness of items in a measurement, a mean-square residual summary statistics, such as Outfit and Infit values, are convenient to use (Wright et al., 1994). Moreover, as the employees' QoL is found to diverge at long commutes (Kroesen, 2022), indoor soundscapes (Torresin et al., 2021; Xiao et al., 2021), physical exercise (Xiao et al., 2021), food intake (Xiao et al., 2021), communication with coworkers (Xiao et al., 2021), children at home (Xiao et al., 2021), distractions while working (Xiao et al., 2021), adjusted work hours (Xiao et al., 2021), and job autonomy (Saragih et al., 2021)–some items may not invariantly measure the employees' QoL between the groups of WFH and WIO. Therefore, if the WHOQOL-BREF is not measurement invariant, the WHOQOL-BREF may be easier for one of the groups to reach a high level of QoL. We therefore, additionally explore the measurement invariance of items in the WHOQOL-BREF in this study.

The purpose of this article is to investigate the employees' QoL under multiple scenarios that have been experienced, including the work modes, the amount of social contact, and the ratio of face-to-face contact. We therefore studied issues regarding (i) whether the employees' QoL varies because of the type of work mode (WFH vs. WIO), the amount of social contact (quantity) (including any kind of contact), and the ratio of face-to-face contact; (ii) whether the employees' QoL is moderated by the number of social contacts and ratio of face-to-face contact given the different work modes; (iii) whether the effect of interaction between the amount of social contact and the ratio of face-to-face contact on the employees' QoL is significant given the different work modes; and (iv) whether the WHOQOL-BREF can invarianctly measure the employees' QoL for different work modes and the number of social contacts.

## Methods

### Design

The data were collected by the Academia Sinica Thematic Research Program in Taiwan with the approval of the Institutional Review Board (IRB) (AS-IRB-HS07-109104). From May 15, 2021, to May 28, 2021, the recruitment was posted on Facebook and Twitter's open pages of several institutions, such as the department of sociology at National Taiwan University, the department of sociology at Academia Sinica, and the Ministry of Foreign Affairs of the Republic of China. The recruitment was also shared on Line and Instagram using snowball sampling. The recruitment focused on the Taiwanese WIO or WFH employees who were over 20 years old and with normal cognitive function. When people click the link on the recruitment, they will be directed to our survey website–Social Distancing Survey. After participants confirmed the electronic informed consent form printed on the website's front page, they started to respond to our survey. The survey included two questionnaires: one basic information questionnaire and the WHOQOL-BREF. In the basic information questionnaire, participants needed to answer a series of questions collected from the International Social Survey Programme (ISSP), 2017 (Sapin et al., 2020). For instance, one was about the amount of their daily average social contacts during the last 2 weeks, namely social contact quantity (including any kind of contact, e.g., chat with, talk to, or text, either face-to-face, by phone, internet, or any other communication device). Four levels were included: C1: 0–4 people, C2: 5–9 people, C3: 10–19 people, and C4: more than 20 people. Next, participants were asked about “the number of contacts that were face-to-face,” which contained two levels: F1: below one half and F2: above one half, called the face-to-face contact ratio. Therefore, participants can further be divided into sixteen groups, resulting from three factors: the type of work mode (WIO vs. WFH), the social contact quantity (C1 vs. C2 vs. C3 vs. C4), and the face-to-face contact ratio (F1 vs. F2), as in a case-control study without experimental manipulation. Additionally, we used the Taiwan version of the WHOQOL-BREF to measure QoL (Yao et al., 2002). This measure contains four domains: physical health (seven items), psychological health (six items), social relationships (three items), and environment (seven items). Individuals that score high on the scale are said to have good QoL. Expected a Posterior (EAP) reliability was 0.84 for the physical health domain, 0.85 for the psychological health domain, 0.73 for the social relationships domain, and 0.78 for the environment domain.

As stated earlier, this study was implemented in Taiwan. Compared to other countries, Taiwan had zero local confirmed cases recorded from April to December 2020 (Hannah et al., 2020), suggesting that Taiwan remarkably succeeded in combating the COVID-19 pandemic. Taiwanese people could, thus, still live normally during the pandemic. However, on May 15, 2021, the number of daily new confirmed cases in Taiwan suddenly rose to triple-digit (1.75 daily new confirmed COVID-19 cases per million people Hannah et al., 2020), forcing the Central Epidemic Command Center (CECC) to raise the warning level to level 3. Then, the outbreak soon reached its peak in late May. During the level three epidemic level alert, the prevention measures included the closure of leisure and entertainment venues, limited religious rituals, and elementary and middle schools were closed (Centers for Disease Control and Prevention, 2021). Besides, following the social distancing rule, many employees were encouraged to work from home. Yet, as Dockery and Bawa (2020) stated, before the COVID-19 outbreak, WFH was more common in “telecommuting” or “teleworking” jobs. Nonetheless, to prevent the company from being terminated altogether, employers needed to make decisions to allow more job positions that may be suitable for WFH to be shifted to WFH, and thus many employees started WFH without previous experience. The sudden change, therefore, presented a challenge to the Tawainese: people needed to re-adapt to this pandemic situation. Hence, this was a valuable time to research the QoL of the WFH employees.

### Statistical analysis

In this article, we researched issues, such as (i) were there differences in the employees' QoL across subgroups of the work mode, the quantity of social contact, and the face-to-face contact ratio? (ii) was the employees' QoL moderated by the social contact quantity and the face-to-face contact ratio given their work mode? (iii) given different work modes, did the employees' QoL vary because of the social contact quantity and the face-to-face contact ratio? (iv) did the WHOQOL-BREF invariantly measure the QoL for subgroups of the work mode, the social contact quantity, and the face-to-face contact ratio? If not, which items in the WHOQOL-BREF measured differently for the work mode, the social contact quantity, and the face-to-face contact ratio? Specifically, the employees' QoL was assessed using the WHOQOL-BREF, fitted, and estimated by the four-dimensional rating scale (MRS) model, the most basic model in Item Response Theory (IRT) for analyzing rating scale items. Plus, given that the method of differential item functioning (DIF) analysis is typically applied to find the difference in the responses of participants among the subgroups, we used the DIF analysis to analyze the responses on the WHOQOL-BREF among subgroups of the work mode, the social contact quantity, and the face-to-face contact ratio using ConQuest software (Adams et al., 2017). Parameters estimated by the DIF analysis are summarized in Table 1. According to the IRT, two sets of parameters are estimated, including person and item parameters. Each parameter belongs to real numbers; the larger the values of person parameters, the higher the QoL, and the greater the values of item parameters, the harder the difficulty (i.e., respondents cannot acquire a high score on a difficult item easily).

**Table 1:** Estimated parameters in the model.

**Symbol**	**Parameter**
**Person parameters**	
(a)	Person ability
(b)	Work mode
(c)	Social contact quantity
(d)	Face-to-face contact ratio
(e)	Work mode × social contact quantity
(f)	Work mode × face-to-face contact ratio
(g)	Social contact quantity × face-to-face contact ratio
(h)	Work mode × Social contact quantity × face-to-face contact ratio
**Item parameters (including item difficulty)**	
(i)	Item difficulty
(j)	Item × work mode
(k)	Item × social contact quantity
(l)	Item × face-to-face contact ratio
(m)	Item × work mode × social contact quantity
(n)	Item × work mode × face-to-face contact ratio
(o)	Item × social contact quantity × face-to-face contact ratio
(p)	Item × work mode × social contact quantity × face-to-face contact ratio

×, interaction effect among the variable terms.

Since the model is complicated, for convenience, we refer to these parameters as (a)–(p) in this article, respectively. The (b)–(d) terms were used to study issue (i). The (e)–(g) terms were used to solve issue (ii). The (h) term was used to investigate issue (iii). If the (a)–(h) terms were significant, the QoL measured by the WHOQOL-BREF differed among the subgroups of the corresponding factor. Besides, the (i)–(l) terms were used to study issue (iv); that is, an item in the (i) term was detected as a problematic item if its Outfit or Infit values were outside the range of [0.6, 1.4] (Wright et al., 1994), showing that most responses of the participants on the item were not in line with expectations. Furthermore, the (j)–(l) terms were tested by Kim et al. (1995)'s method for detecting DIF items. As DIF indicates that an item measures differently for one subgroup of a population than others, an item detected as a DIF item for one of the factors demonstrates an association between the item and the corresponding factor.

## Results

### Demographic characteristics

As shown in Table 2, the total number of participants was 1,391; 588 were the WIO and 803 were the WFH employees. Among the two subgroups, female participants were more than male participants. Many participants were single (81%) with an educational level of college and above (93%). The average age of the participants was 32.75 and 29.37 years for the WIO and WFH employees, respectively.

**Table 2:** Demographic characteristics of 1,391 participants.

**Characteristic**	**Work mode**	
	**WIO**	**WFH**
Overall	588	803
**Gender**		
Female	399	569
Male	189	234
**Age**		
Mean	32.75	29.37
SD	13.13	9.60
**Marital status**		
Married	133	125
Single	455	678
**Education**		
Elementary	1	0
Middle	3	1
High	73	21
College and above	511	781

WIO, work in the office; WFH, work from home.

### Main effects

**Figure 1:**
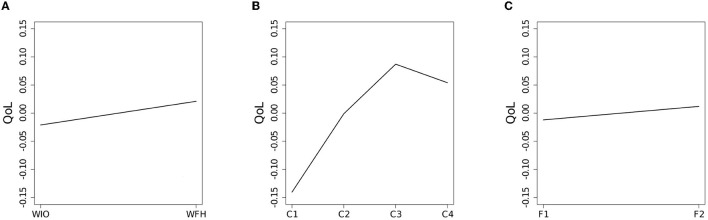
The mean scores of QoL for **(A)** the work mode, **(B)** the social contact quantity, and **(C)** the face-to-face contact ratio.

There was a significant difference in the QoL between the WIO and WFH employees [the (b) term] [$\chi_{\left(1\right)}^{2}=8.52,\;p=0.0035$]; that is, the WIO employees scored lower on the overall domains of QoL than the WFH employees as plotted in Figure 1A. Additionally, the difference in QoL among the levels of social contact quantity [the (c) term] was statistically significant [$\chi_{\left(3\right)}^{2}=209.38,\;p<0.001$]. As shown in Figure 1B, the employees' QoL increased as the amount of social contact increased, except for the last level, C4. Moreover, as shown in Figure 1C, the difference in QoL between the ratios of face-to-face contact was not statistically significant [$\chi_{\left(1\right)}^{2}=2.71,\;p=0.0997$], and thus the result was retained.

### Two-way interaction effects

**Figure 2:**
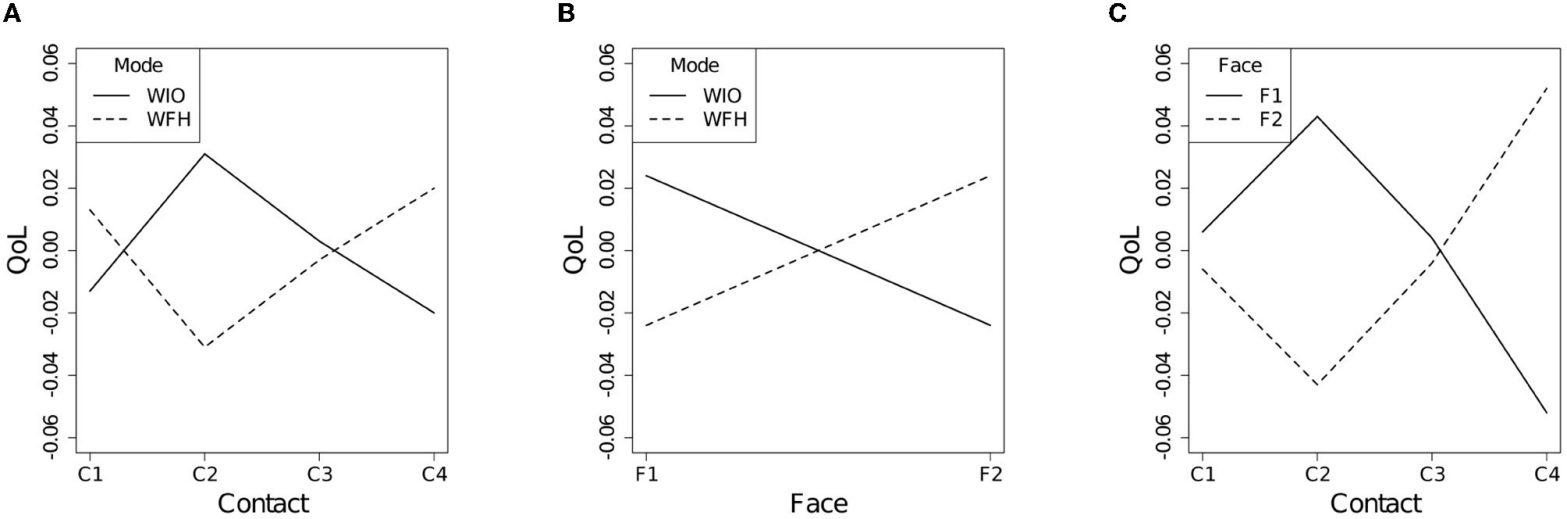
Interaction plots **(A)** between the work mode and the social contact quantity, **(B)** between the work mode and the face-to-face contact ratio, and **(C)** between the social contact quantity and the face-to-face contact ratio.

The interaction effect between the work mode and the social contact quantity [the (e) term] was significant [$\chi_{\left(3\right)}^{2}=10.89,\;p=0.012$]. As shown in Figure 2A, for the WFH employees, their QoL increased as their social contact quantity increased. Yet, their QoL decreased at the C2 level. Additionally, the interaction effect between the work mode and the face-to-face contact ratio [the (f) term] was significant [$\chi_{\left(1\right)}^{2}=11.39,\;p<0.001$]. As plotted in Figure 2B, for the WFH employees, their QoL increased as the face-to-face contact ratio increased. Additionally, the interaction effect between the social contact quantity and the face-to-face contact ratio [the (g) term] was significant. As demonstrated in Figure 2C, when over half of the social contacts were face-to-face, the employees QoL increased as the social contact quantity increased. However, the QoL decreased at the C2 level.

### Three-way interaction effects

**Figure 3:**
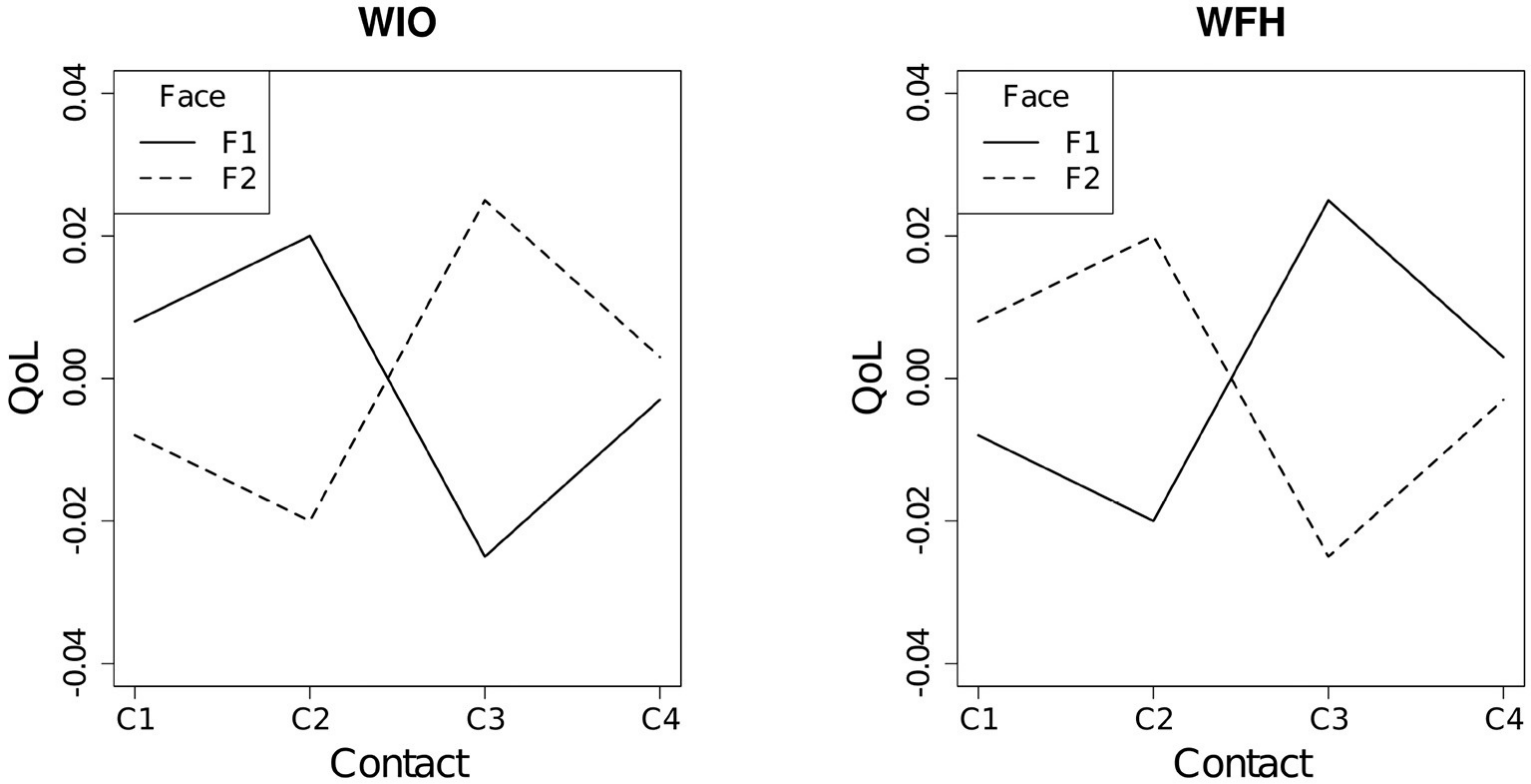
Interaction plot between the social contact quantity and the face-to-face contact ratio for the WIO **(left)** and WFH **(right)** employees.

The three-way interaction effect among the type of work modes, the social contact quantity, and face-to-face contact ratio [the (h) term] was significant [$\chi_{\left(3\right)}^{2}=9.07,\;p=0.028$]. As shown in Figure 3, the difference in the QoL at the C4 level of social contact quantity between the F1 and F2 levels was the lowest. Furthermore, for the WIO employees, when over half of the social contacts were face-to-face (F2), the QoL decreased at the C1 and C2 levels of social contact quantity but increased at the C3 level. On the contrary, for the WFH employees, when over half contacts were face-to-face contacts (F2), the QoL increased as social contact quantity increased from the C1 to C2 level. However, the QoL decreased at the C3 level for F2.

### Item analysis

First, items 3 (pain), 4 (medication), 15 (mobility), and 19 (esteem) were detected as problematic items, since their Outfit and Infit values were out of the range of [0.6, 1.4]: the Outfit and Infit values for item 3 (pain) were 2.07 and 2.01, item 4 (medication) were 1.79 and 1.88, item 15 (mobility) were 1.63 and 1.69, and item 19 (esteem) were 0.57 and 0.57. These values indicated that items 3 (pain), 4 (medication), and 15 (mobility) were underfitted, and item 19 (esteem) was overfitted by the MRS model.

Additionally, many items were detected as biased in measuring QoL among the subgroups of the work mode, the social contact quantity, and the face-to-face contact ratio as demonstrated in Tables 3–**5**. As shown in Table 3, for the work mode, items 3 (pain), 4 (medication), 10 (energy), 15 (mobility), and 17 (activities) in the physical health domain; item 6 (meaningful life) in the psychological health domain; item 21 (sex life) in the social relationships domain; and items 9 (environment), 13 (information), and 14 (leisure) in the environment domain were detected as DIF items. Specifically, for the WFH employees, items 3 (pain), 15 (mobility), 21 (sex life), and 14 (leisure) were harder, while the others were easier.

**Table 3:** Summary table for the DIF analysis in the items of WHOQOL-BREF across subgroups of the work mode.

**Domain**	**Number**	**Facet**	**Work mode**		$\chi_{\left(1\right)}^{2}$	**Significance**
			**WIO**	**WFH**		
PHY	3R	Pain	–0.05	0.05	8.00	**
PHY	4R	Medication	0.07	–0.07	13.22	***
PHY	10	Energy	0.04	–0.04	5.56	*
PHY	15	Mobility	–0.11	0.11	36.64	***
PHY	16	Sleep	0.01	–0.01	0.17	-
PHY	17	Activities	0.04	–0.04	5.64	*
PHY	18	Work	–0.01	0.01	0.02	-
PSY	5	Positive feelings	0.02	–0.02	1.84	-
PSY	6	Meaningful life	0.04	–0.04	5.28	*
PSY	7	Think	–0.02	0.02	1.12	-
PSY	11	Body image	–0.02	0.02	1.39	-
PSY	19	Esteem	–0.01	0.01	0.42	-
PSY	26R	Negative feelings	–0.01	0.01	0.14	-
SOC	20	Personal relationships	0.00	0.00	0.00	-
SOC	21	Sex life	–0.05	0.05	7.07	**
SOC	22	Friends support	0.05	–0.05	3.76	-
ENV	8	Safety	–0.02	0.02	1.16	-
ENV	9	Environment	0.07	–0.07	16.59	***
ENV	12	Finances	0.02	–0.02	1.41	-
ENV	13	Information	0.08	–0.08	18.90	***
ENV	14	Leisure	–0.10	0.10	35.28	***
ENV	23	Home	0.02	–0.02	0.92	-
ENV	24	Health services	–0.01	0.01	0.36	-
ENV	25	Transport	–0.06	0.06	1.50	-

R, reverse-coded item; PHY, physical health; PSY, psychological health; SOC, social relationships; ENV, environment. Significance levels are indicated as follows:

*** *p* < 0.001,

** *p* < 0.01, and

* *p* < 0.05.

Similarly, for the social contact quantity, as shown in Table 4, items 16 (sleep) and 18 (work) in the physical health domain; items 5 (positive feelings), 6 (meaningful life), 7 (think), and 11 (body image) in the psychological health domain; items 20 (personal relationships) and 21 (sex life) in the social relationships domain; item 9 (environment) in the environment domain were detected as DIF items. Specifically, the difficulty of items 18 (work), 6 (meaningful life), 7 (think), and 20 (personal relationships) lowered as the amount of social contact increased. At the same time, the difficulty of the other DIF items rose following the increased amount of social contact.

**Table 4:** Summary table for the DIF analysis in the items of the WHOQOL-BREF across subgroups of social contact quantity.

**Domain**	**Number**	**Facet**	**Social contact quantity**				$\chi_{\left(3\right)}^{2}$	**Significance**
			**C1**	**C2**	**C3**	**C4**		
PHY	3R	Pain	–0.05	–0.04	0.06	0.03	5.68	-
PHY	4R	Medication	–0.10	–0.04	0.01	0.13	7.75	-
PHY	10	Energy	0.01	–0.05	0.06	–0.02	4.85	-
PHY	15	Mobility	0.03	0.01	–0.06	0.02	3.14	-
PHY	16	Sleep	–0.10	–0.06	–0.04	0.20	16.98	***
PHY	17	Activities	0.01	0.04	–0.07	0.03	4.55	-
PHY	18	Work	0.20	0.15	0.05	–0.39	10.52	*
PSY	5	Positive feelings	–0.06	–0.08	–0.05	0.19	14.47	**
PSY	6	Meaningful life	0.11	0.00	–0.06	–0.06	11.67	**
PSY	7	Think	0.00	0.14	0.00	–0.14	20.5	***
PSY	11	Body image	–0.08	–0.13	0.06	0.15	24.62	***
PSY	19	Esteem	0.02	0.06	0.01	–0.09	4.53	-
PSY	26R	Negative feelings	0.01	0.00	0.03	–0.04	0.19	-
SOC	20	Personal relationships	0.10	0.09	0.04	–0.22	19.32	***
SOC	21	Sex life	–0.08	–0.10	0.05	0.13	15.46	**
SOC	22	Friends support	–0.02	0.01	–0.09	0.09	2.92	-
ENV	8	Safety	0.02	0.03	0.04	–0.09	2.74	-
ENV	9	Environment	–0.19	–0.05	0.01	0.22	33.09	***
ENV	12	Finances	0.02	–0.03	–0.03	0.04	1.95	-
ENV	13	Information	0.07	–0.02	–0.07	0.02	6.06	-
ENV	14	Leisure	0.03	0.02	–0.01	–0.04	1.07	-
ENV	23	Home	–0.01	–0.01	0.09	–0.06	5.15	-
ENV	24	Health services	0.01	0.03	0.05	–0.10	3.31	-
ENV	25	Transport	0.05	0.04	–0.08	–0.01	0.84	-

R, reverse-coded item; PHY, physical health; PSY, psychological health; SOC, social relationships; ENV, environment. Significance levels are indicated as follows:

*** *p* < 0.001,

** *p* < 0.01, and

* *p* < 0.05.

For the face-to-face contact ratio, items 3 (pain) and 4 (medication) in the physical health domain; items 5 (positive feelings), 19 (esteem) in the psychological health domain; and items 13 (information) and 14 (leisure) in the environment domain were detected as DIF items as shown in Table 5. To be specific, items 4 (medication), 5 (positive feelings), and 13 (information) were more difficult for employees who had over half of their social contacts as face-to-face (F2).

**Table 5:** Summary table for the DIF analysis in the items of the WHOQOL-BREF across subgroups of the face-to-face contact ratio.

**Domain**	**Number**	**Facet**	**Face contact ratio**		$\chi_{\left(1\right)}^{2}$	**Significance**
			**F1**	**F2**		
PHY	3R	Pain	0.04	–0.04	5.56	*
PHY	4R	Medication	–0.12	0.12	39.22	***
PHY	10	Energy	–0.02	0.02	1.68	-
PHY	15	Mobility	0.03	–0.03	3.08	-
PHY	16	Sleep	0.00	0.00	0.01	-
PHY	17	Activities	0.00	0.00	0.08	-
PHY	18	Work	0.07	–0.07	2.71	-
PSY	5	Positive feelings	–0.05	0.05	10.12	**
PSY	6	Meaningful life	0.01	–0.01	0.68	-
PSY	7	Think	0.02	–0.02	1.39	-
PSY	11	Body image	–0.02	0.02	1.68	-
PSY	19	Esteem	0.04	–0.04	5.84	*
PSY	26R	Negative feelings	0.00	0.00	0.00	-
SOC	20	Personal relationships	0.03	–0.03	3.70	-
SOC	21	Sex life	–0.02	0.02	1.55	-
SOC	22	Friends support	–0.01	0.01	0.28	-
ENV	8	Safety	–0.02	0.02	1.55	-
ENV	9	Environment	–0.03	0.03	2.16	-
ENV	12	Finances	–0.01	0.01	0.32	-
ENV	13	Information	–0.06	0.06	9.55	**
ENV	14	Leisure	0.06	–0.06	10.76	**
ENV	23	Home	–0.01	0.01	0.12	-
ENV	24	Health services	0.03	–0.03	2.16	-
ENV	25	Transport	0.04	–0.04	0.75	-

R, reverse-coded item; PHY, physical health; PSY, psychological health; SOC, social relationships; ENV, environment. Significance levels are indicated as follows:

*** *p* < 0.001,

** *p* < 0.01, and

* *p* < 0.05.

## Discussion

To evaluate the QoL of the employees in a workplace, there is need to additionally view the employees' work performance as a whole as opposed to only focusing on their physical or psychological health (Rabianski, 2007). Measuring the QoL can therefore provide organizational management professionals with valuable insights into the lives of employees. The pandemic has spread widely since early 2020 and has lead to hundreds and thousands of deaths. To lower the transmission of the virus, governments implemented the WFH policy with regards to employees, as opposed to WIO, to maintain and promote the social distancing rule. In this study, we researched the employees' QoL during the COVID-19 pandemic. We further assessed the differences in the employees' QoL, measured by the WHOQOL-BREF, across subgroups depending on the type of work mode (WFH vs. WIO), the amount of social contact (quantity), and the ratio of face-to-face contact. These comparisons were made to understand the factors affecting the employees' QoL during the pandemic.

Previous studies indicated that the WFH policy was associated with mental illness as a result of the social-distancing rule (Marroquín et al., 2020). However, through social support (Mariani et al., 2020; Qi et al., 2020) and non-face-to-face contact (Litwin and Levinsky, 2022), individual's mental problems were improved during the pandemic. Although WFH was beneficial in maintaining social distancing among people, this study's findings indicated that the WFH employees' QoL was lower than the WIO employees' QoL when the WFH employees' social contact quantity was low (contacted 5–9 people average a day)–this was consistent with the previous studies. Nevertheless, the WFH employees' QoL improved and became superior to the WIO employees' QoL as their social contact quantity increased. However, the WFH employees' QoL improved when most social contacts were experienced face-to-face. The social connections and face-to-face contact were necessary for employees to maintain a good QoL. An increased amount of face-to-face contact enhanced the employees' QoL. However, while considering the three-way interactions, we further found that the WFH employees' QoL decreased as the number of social contacts increased when most were experienced as face-to-face contacts. On the contrary, the WIO employees' QoL rose as the number of face-to-face contacts increased. Therefore, according to our findings, for the WFH employees, having social contact did not always increase their QoL, as it was moderated by the ratio of face-to-face contact. Interestingly, several studies indicated that the use of the internet undoubtedly lowered adverse life events and promoted social support (LaRose et al., 2001; Amichai-Hamburger and Ben-Artzi, 2003; Lewandowski et al., 2011). However, in improving mental health, face-to-face contact had a more significant effect as opposed to non-face-to-face contact (Achterhof et al., 2022; Fujiwara et al., 2022). In contrast, in improving social quality, interactions that took place online were better than face-to-face contact (Achterhof et al., 2022), which may be explained by Gonzales (2014)'s findings that exhibit that text-based communication was more influential than face-to-face communication on self-esteem and further determined meaningful social interactions. Therefore, those may indicate that the WFH employees' QoL was more affected by social support, and as a result the WFH employees' QoL improved as the number of non-face-to-face contacts increased.

In assessing the QoL, the WHOQOL-BREF was undoubtedly well developed. However, when analyzed by the type of work mode, the social contact quantity, and the face-to-face contact ratio, many items in these three indicated a bias; as items 3 (pain), 4 (medication), 15 (mobility), and 19 (esteem) were detected as problematic items as a result of their Outfit and Infit values which were outside the criteria of 0.6 and 1.4. More specifically, items 3 (pain), 4 (medication), and 15 (mobility) were underfitted as their Outfit and Infit values were >1.4. In terms of items 3 and 4, as they were reverse-coded items, their meaning could diverge as a result of the positive and negative wording (Roskam, 1985), further resulting in a dimensionality problem (Herche and Engelland, 1996). However, the reason for item 15 (mobility) to be seen as a problematic item may be a result of its content seeming ambiguous to the WFH employees; as its content “the ability to get around” was connected to the situation of WFH, while also measuring disability, and therefore being detected as a DIF item. In addition, for item 19 (esteem), the satisfaction with myself, because many people responded to this item in the middle, their responses may not have been consistent with their QoL, and as a result this item was measured with bias.

Additionally, many items were detected as DIF items associated with the type of work mode, the social contact quantity, and the face-to-face contact ratio. In terms of the type of work mode, the WFH employees were found to have higher performance on items 4 (medication), 10 (energy), 17 (activities), 6 (meaningful life), 9 (environment), and 13 (information) as opposed to the WIO employees, indicating that the WFH employees tend to be satisfied in these facets. At the same time, items 3 (pain), 5 (mobility), 21 (sex life), and 14 (leisure) were more difficult for the WFH employees than for the WIO employees. These finding are consistent with Guler et al. (2021)'s study which stated that the WFH employees tend to suffer from physical pains (e.g., low back pain and weight gain) as outdoor activities decreased while they were more productive. However, the WFH employees were more satisfied with their physical environment, as they had more control of the environmental factors (Salamone et al., 2021; Xiao et al., 2021). The sexual intimacy did not increase despite couples being stuck at home as Matchan (2020) and Salisbury (2020) proposed, however, it decreased in line with Prabowo et al. (2022)'s findings, probably as a result of the anxiety caused by the pandemic (Liu et al., 2010). These DIF items indicated the drawbacks and benefits of the WFH mode as opposed to the WIO mode with regards to the QoL.

Similarly, in terms of the social contact quantity, following the increased amount of social contact, the difficulty of items 18 (work), 6 (meaningful life), and 20 (personal relationship) decreased. Therefore, employees with a larger social contact quantity tended to be satisfied with these facets. On the contrary, the difficulty of items 16 (sleep), 5 (positive feelings), 11 (body image), 21 (sex life), and 9 (environment) increased as the amount of social contact increased. This was consistent with the finding that social contact is an essential aspect of the perceived quality of work (Jones et al., 2017). Additionally, women tend to be anxious when their social contact quantity increased (Tiggemann and Slater, 2017). Despite this, our findings exhibited that employees' sleep quality was disrupted when their social contact quantity increased. There was no doubt that social isolation and loneliness were linked to sleep issues (Pressman et al., 2005; Yu et al., 2018). However, as a result of the network size not predicting loneliness (Pressman et al., 2005; Kim and Shen, 2020), there may be some confounding variables moderating the relationship. For instance, the relationship quality was associated to sleep quality; as positive ties promoted sleep quality, while adverse ties lowered sleep quality (Kent et al., 2015), Additionally, the size of the social network could predict the level of life satisfaction, but, for older people, a social network which mainly composed of close friends predicted that the level of life satisfaction was higher (Chang et al., 2015; Kim and Shen, 2020). Therefore, the type and quality of social contacts would need to be considered in future studies. In addition, the relationships between the quantity of social contact and a meaningful life, sex life, and the environment are still unknown and therefore need to be explored.

Moreover, in terms of the face-to-face contact ratio, items 4 (medication), 5 (positive feeling), and 13 (information) were harder for employees whose social contacts were mainly face-to-face. On the contrary, items 3 (pain), 19 (esteem), and 14 (leisure) were easier for employees whose social contacts were mostly face-to-face. This suggests that employees with a high ratio of face-to-face contact tend not to be satisfied with life and suffer from physical pains and needed medical treatment to function in their daily lives. Intuitively, this was connected to the pandemic: people with a high ratio of face-to-face contact tend to be infected by the virus. Nevertheless, the relationship between face-to-face contact ratio and the availability of information, esteem, and leisure, were unknown, which needs further consideration in future studies. In summary, the WHOQOL-BREF will need several revisions for measuring the QoL among subgroups with regards to the type of work mode and the social contact quantity, in further studies as many items were found to be correlated with the type of work mode, the quantity of social contact, and the face-to-face contact ratio. Nevertheless, by measuring the QoL using the WHOQOL-BREF, we found the facets where subgroups depending on the type of work mode, the quantity of social contact, and the face-to-face contact ratio which had a divergent performance based on which researchers can conduct further studies for studying the employees' QoL.

In summary, the WFH mode is a choice that is made with an attempt to maintain social distancing among people in the pandemic. In this study, we found that the WFH employees' QoL was higher than the WIO employees' QoL. Despite this, the WFH employees' QoL was moderated by social contacts; as the WFH employees' QoL decreased as social contact decreased which resulted in the WFH employees' QoL being lower than the WIO employees' QoL depending on whether the amount of social contact was minimal. It is therefore important to think about how to keep people connected in order to maintain job performance. There were many DIF items that indicated an association between the employees' QoL and the work mode or the social contact quantity, exhibiting that the WHOQOL-BREF could not invariantly measure the employees with different type of work modes and the amount of social contact. Nevertheless, these items provided valuable insight into the factors affecting the employees' QoL.

This study experienced limitations. For instance, we conducted this study in Taiwan, so the association among the items of WHOQOL-BREF and the different types of work mode, the quantity of social contact, and the face-to-face contact ratio may not be generalized to other countries. In addition, owing to the fact that this study's participants were recruited on social media, they may not be representative of the general population; they may tend to have greater access to the internet. Despite this, it demonstrated the scenario that many people were forced to WFH without having previous experience of doing so. We failed to consider controlling the job category. Owing to the fact that not every job is suitable for the WFH mode, the influence of the WFH experience on the QoL may also be moderated by the job category. In addition, as highly educated and high-income people were much more likely to shift to WFH (Bick et al., 2020) but not the lower skilled, lower paid (Dockery and Bawa, 2020), poorly educated people, and those on temporal contracts (Garrote Sanchez et al., 2021)—the characteristics determining the suitability of WFH may also be the mediator.

## Data availability statement

The raw data supporting the conclusions of this article are available by contacting the corresponding author.

## Ethics statement

The studies involving human participants were reviewed and approved by Academia Sinica (ASIRB-HS07-109104). The patients/participants provided their written informed consent to participate in this study.

## Author contributions

The first draft of the manuscript was written by C-HL. All authors commented on previous versions of the manuscript, contributed to the study conception and design, performed material preparation, data collection and analysis, read, and approved the final manuscript.
